# Fucoidan partly prevents CCl_4_-induced liver fibrosis

**DOI:** 10.1016/j.ejphar.2007.11.015

**Published:** 2008-02-12

**Authors:** Shinji Hayashi, Ayano Itoh, Katsuhiro Isoda, Masuo Kondoh, Masaya Kawase, Kiyohito Yagi

**Affiliations:** Laboratory of Bio-Functional Molecular Chemistry, Graduate School of Pharmaceutical Sciences, Osaka University, Osaka 565-0871, Japan

**Keywords:** Fucoidan, Liver fibrosis, Hepatocyte, Hepatic stellate cell

## Abstract

Fucoidan, a sulfated polysaccharide extracted from brown algae, has a wide range of biological activities, including anti-inflammatory, anti-viral, and anti-tumor activities. In the present study, we investigated the effects of fucoidan on CCl_4_-induced liver fibrosis. Administration of fucoidan reduced CCl_4_-induced acute and chronic liver failure. Hepatic fibrosis induced by CCl_4_ was also attenuated by injection of fucoidan. Damage to hepatocytes and activation of hepatic stellate cells are key events in liver fibrosis, and, interestingly, treatment of hepatocytes with fucoidan prevented CCl_4_-induced cell death and inhibited the proliferation hepatic stellate cells. These results indicate that fucoidan might be a promising anti-fibrotic agent possessing dual functions, namely, protection of hepatocytes and inhibition of hepatic stellate cell proliferation.

## 1 Introduction

Hepatic fibrosis results from chronic damage to the liver in conjunction with the progressive accumulation of fibrillar extracellular matrix proteins (Friedman, 1993; Gressner, 1995; Lieber, 1999). The main causes of liver fibrosis are infection with hepatitis B or C, alcohol abuse, and non-alcohol steatohepatitis. There are over 100 million people with hepatic fibrosis in the world.

The liver consists of parenchymal (hepatocytes) and non-parenchymal cells (Kupffer, stellate, and endothelial cells). Hepatic fibrosis is triggered by specific intercellular interactions among some of these cells. Kupffer cells are activated by membrane components from damaged hepatocytes and infiltrating inflammatory cells. The activated Kupffer cells release pro-fibrotic factors, such as transforming growth factor-β, reactive oxygen species, and other factors (Wu et al., 1998). These pro-fibrotic factors act on hepatic stellate cells, which are key players in hepatic fibrosis (Gressner, 1995).

Hepatic stellate cells are normally quiescent and produce only small amounts of extracellular matrix components, such as laminin and collagen type IV, during the formation of basement membrane (Maher and Bissell, 1993). Exposure of hepatic stellate cells to the pro-fibrotic factors activates hepatic stellate cells, leading to a changed to a myofibroblast phenotype and an increase in the production of collagen (Friedman, 1999; Geerts, 2001). Preventing the activation of hepatic stellate cells is thus a promising anti-fibrotic strategy. Indeed, administration of antioxidants has been shown to suppress the activation of hepatic stellate cells, thereby preventing liver fibrosis, and inhibition of hepatocyte cell death can reduce liver fibrosis (Houglum et al., 1997; Maher et al., 1997; Horie et al., 2003; Canbay et al., 2002; Song et al., 2003).

Brown algae seaweeds contain both soluble dietary fiber polysaccharides, including alginates, fucans, and laminarans, and insoluble fibers, which are mostly composed of cellulose (Lahaye and Kaeffer, 1997; Kloareg and Quatrano, 1988). The fucans are cell wall polysaccharides that contain variable amounts of fucose, uronic acids, galactose, xylose, and sulfate. They are classified in three types according to their chemical composition: fucoidan, ascophyllan, and glucuronofuco-galactan sulfate (Lahaye and Kaeffer, 1997; Kloareg and Quatrano, 1988; Mabeau et al., 1990). Fucoidan is a complex sulfated polysaccharide derived from *Fucus vesiculosus*, and it has a variety of biological activities, including anti-inflammatory, anti-viral, anti-liver failure, and anti-tumor activities (Boisson-Vidal et al., 1995; Saito et al., 2006). Furthermore, fucoidan interacts with transforming growth factor-β and has antioxidative properties (Xue et al., 2001; McCaffrey et al., 1994). These findings indicate that fucoidan may have anti-fibrotic activity, but whether fucoidan can attenuate hepatic fibrosis is unknown. Therefore, in the present study, we evaluated the effects of fucoidan on hepatic injury and fibrosis. We found that fucoidan may be useful as a novel type of anti-fibrotic agent.

## 2 Materials and methods

### 2.1 Reagents

CCl_4_ was purchased from Wako Pure Chemicals, Co. Ltd. (Osaka, Japan). Fucoidan was obtained from Sigma Chemical, Co. Ltd. (St. Louis, MO). CCl_4_ and fucoidan were dissolved in olive oil and saline in animal experiment, respectively. In cellular experiments, fucoidan was dissolved in cultured medium for hepatocytes or hepatic stellate cells as described below. CCl_4_ was dissolved in dimethylsulfoxide at 100 mM.

### 2.2 Animals and experimental protocols

All of the experimental protocols conformed to the ethics guidelines of the Graduate School of Pharmaceutical Sciences, Osaka University. Male Sprague–Dawley rats (200–250 g) and male ddy mice (6 weeks old) were obtained from SLC (Shizuoka, Japan). The mice were housed in an environmentally controlled room (lights on from 8:00 to 20:00; temperature, 23 ±1.5 °C). Animals had free access to water and commercial chow (Type MF, Oriental Yeast, Tokyo, Japan).

In the acute liver injury model, CCl_4_ was intraperitoneally administrated to mice at 0.3 ml/kg body weight with or without intravenous injection of fucoidan (25 and 50 mg/kg body weight). After 24 h of fucoidan injection, the mice were anesthetized, and the blood was recovered. In the chronic liver injury model, CCl_4_ (0.3 ml/kg body weight) and fucoidan (50 mg/kg body weight) were administered orally and intravenously, respectively, to mice twice a week for 8 weeks. The liver and blood were recovered from the mice under anesthesia. The serum was separated from the blood and stored at − 20 °C before assay.

### 2.3 Aspartate aminotransferase (AST) and alanine aminotransferase (ALT) assays

Serum AST and ALT levels were measured using commercially available kits (Mitsubishi Kagaku Iatron Inc., Tokyo, Japan) according to the manufacturer's instructions.

### 2.4 Analysis of fibrosis

Liver specimens were fixed with 10% formaldehyde and embedded in paraffin. Tissue sections were mounted on slides, and Azan staining was performed to analyze the extent of fibrosis. After establishing a background for each micrograph, the number of pixels showing a blue color (stained collagen fibers) was determined with Scion Image (National Institutes of Health, Bethesda, MD), and the percentage of fibrosis in the liver was calculated as the ratio of the blue-colored area to the total area of the liver.

### 2.5 Isolation of hepatocytes and assay of viability

Hepatocytes were isolated from rats by perfusion of the liver with collagenase (Seglen, 1976). The cells were cultured in William's medium E containing 1 nM insulin, 1 nM dexamethasone, and 10% fetal bovine serum. The cells were seeded onto a dish at 1 × 10^5^ cells/cm^2^ in the absence or presence of fucoidan at 0, 0.3, 0.5, and 1.0 mg/ml. After 6 h of treatment with fucoidan, CCl_4_ was added to the cells at 1 mM. After 3 h, the viability of the cells was assayed by staining with Trypan blue.

### 2.6 Isolation of hepatic stellate cells and assay of viability

Hepatic stellate cells were isolated from rat livers by perfusion with collagenase and pronase, followed by centrifugation on a Nycodenz cushion as described previously (Kawada et al., 1993). The cells were cultured in Dulbecco's modified Eagle's medium supplemented with 10% fetal bovine serum. The cells were then seeded onto a dish at 5 × 10^5^ cells/cm^2^ and then treated with fucoidan at 0, 0.3, 0.5, and 1.0 mg/ml for 24 and 48 h. The viability of the cells was determined by mitochondrial conversion of 3-(4, 5-dimethylthiazol-2-yl)-2,5-diphenyltetrazolium bromide (Mosmann, 1983). The viability of the cells was calculated compared to cells at 0 h (i.e., prior to treatment with fucoidan).

## 3 Results

### 3.1 Effect of fucoidan on acute liver injury

**Fig. 1:**
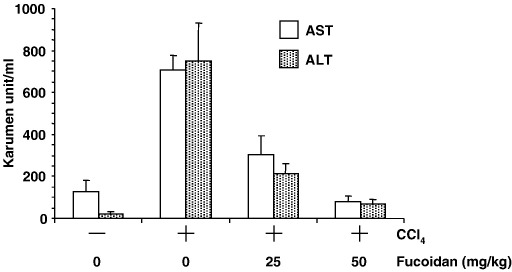
Effect of fucoidan on CCl_4_-induced hepatic injury. Mice received intraperitoneal injection of CCl_4_ and intravenous injection of fucoidan. After 24 h, blood was recovered, and the serum AST (open column) and ALT (slashed column) levels were determined using commercially available kits. Results represent means ± S.D. (*n* = 4).

We first examined the effect of fucoidan on acute liver failure induced by single injection of CCl_4_. Intraperitoneal injection of mice with CCl_4_ increased biochemical markers of liver injury. Specifically, 24 h after injection of CCl_4_, serum AST and ALT had increased from 125 to 707 and from 21 to 752 karumen unit/ml, respectively (Fig. 1). Intravenous administration of fucoidan attenuated this elevation of serum AST and ALT (304 and 214 karumen unit/ml, respectively, at 25 mg/kg fucoidan). Notably, injection of 50 mg/kg fucoidan restored serum AST and ALT to normal levels (77 and 67 karumen unit/ml, respectively). Together, these results indicate that fucoidan is a potent inhibitor of acute CCl_4_-induced liver injury.

### 3.2 Effect of fucoidan on chronic liver injury

**Fig. 2:**
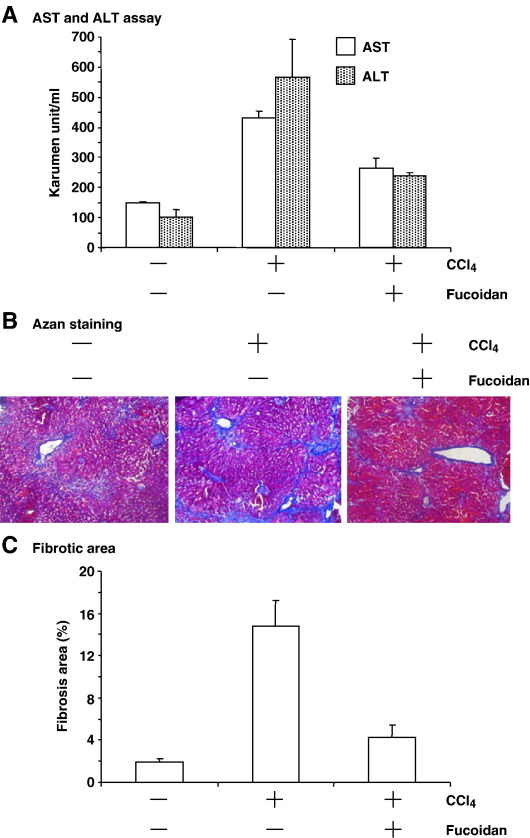
Effect of fucoidan on CCl_4_-induced chronic hepatic injury. Mice were injected orally with CCl_4_ (0.3 ml/kg body weight) and intravenously with fucoidan (50 mg/kg body weight) twice a week for 8 weeks. (A) Blood was recovered for determination of serum AST (open column) and ALT (slashed column). Results indicate are means ± S.D. (*n* = 4). (B) Livers were recovered, sectioned, and stained with Azan. Blue and red areas correspond to fibrotic and normal regions, respectively. (C) The ratio of the fibrotic blue area to the blue and red area of the liver was calculated from the sections using NIH Scion Image. The calculation was performed on five images. Results represent means ± S.D. (*n* = 4).

To evaluate the effect of fucoidan on chronic liver injury, we continuously administered CCl_4_ to mice twice a week for 8 weeks. Serum AST and ALT levels increased from 149 to 433 and from 101 to 568 karumen unit/ml, respectively (Fig. 2A). Azan staining revealed that collagen accumulated in the liver, indicating the onset of liver fibrosis (Fig. 2B). Indeed, the fibrotic area in the liver increased from 1.9% to 14.8% of the whole liver (Fig. 2C). The elevation of AST and ALT levels was significantly reduced by administration of fucoidan (from 433 to 265 and from 568 to 238 karumen unit/ml, respectively; Fig. 2A). The increase in fibrotic area induced by CCl_4_ was also attenuated by fucoidan (from 14.8% to 4.3%; Fig. 2C). Together, these findings show that fucoidan may be useful for treatment of hepatic fibrosis in chronic liver injury.

### 3.3 Effect of fucoidan on hepatocytes and stellate cells

**Fig. 3:**
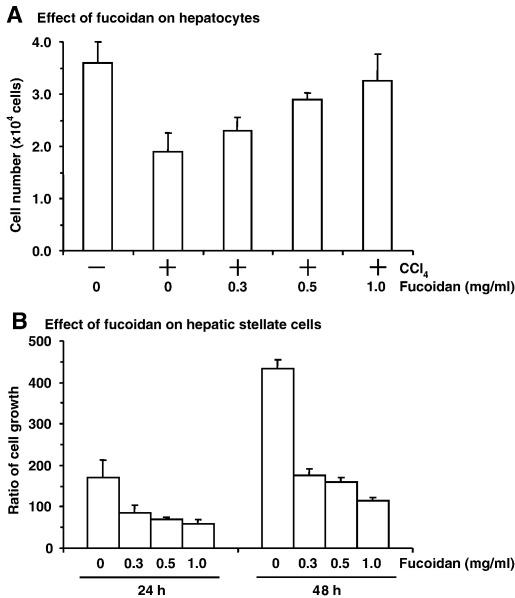
Effect of fucoidan on CCl_4_-treated hepatocytes and hepatic stellate cells. (A) Effect of fucoidan on CCl_4_-induced cell death in hepatocytes. Hepatocytes were treated with CCl_4_ (1.0 mg/ml) in the absence or presence of fucoidan at the indicated concentration for 3 h. Viable cells were counted by Trypan blue staining. Results represent means ± S.D. (*n* = 4). (B) Effect of fucoidan on growth of hepatic stellate cells. Hepatic stellate cells were treated with fucoidan at the indicated concentration for 24 and 48 h. The viability of the cells was assayed by mitochondrial conversion of 3-(4,5-dimethylthiazol-2-yl)-2,5-diphenyltetrazolium bromide. The growth ratio was calculated as a percentage of the viability at 0 h. Results represent means ± S.D. (*n* = 4).

As described above, our preliminary results indicated that fucoidan may be useful for the treatment of hepatic fibrosis. During the initiation of hepatic fibrosis, hepatocytes release a paracrine factor that stimulates hepatic stellate cell growth, leading to the damage of hepatocyte membranes (Gressner, 1995; Gutierrez-Reyes et al., 2007). Therefore, to determine how fucoidan prevents liver injury, we evaluated its effects on hepatocytes and hepatic stellate cells. As shown in Fig. 3A, treatment of hepatocytes with CCl_4_ (1 mM) reduced their viability to 63.3% of control (untreated) cells. In addition, fucoidan dose-dependently reduced CCl_4_-induced cell death, with complete prevention of cell death at 1 mg/ml. Alone, fucoidan did not show any cytotoxicity at 1.0 mg/ml in hepatocytes (data not shown). CCl_4_-induced cell death was not inhibited by pre-incubation of CCl_4_ with fucoidan (data not shown), indicating that the protective effects of fucoidan on CCl_4_-induced cell death is not due to absorption of CCl_4_ by fucoidan. In contrast, the viability of hepatic stellate cells was reduced to 26.4% of the control by a 48-h treatment with 1.0 mg/ml fucoidan (Fig. 3B). Thus, both the protective effects of fucoidan against CCl_4_-induced cell death in hepatocytes and its cytotoxicity to hepatic stellate cells might contribute to its anti-fibrotic activity.

## 4 Discussion

Fucoidan, the sulfated polysaccharides of brown algae, contains l-fucose residues as the main sugar constituent along with sulfate esters. Although fucoidan is known to have many biological activities, including anti-coagulant, anti-thrombosis, anti-inflammatory, anti-liver failure, and anti-tumor activities (Boisson-Vidal et al., 1995; Berteau and Mulloy, 2003; Saito et al., 2006), this is the first investigation of its anti-fibrotic activity. Our results show for the first time that fucoidan can reduce hepatic fibrosis in an animal model.

Studies in animal models of hepatic fibrosis show that extracellular matrix components accumulate in interstitial regions of the liver around central veins or in the portal tracts. Normally, hepatic stellate cells exist in a quiescent state, but they become activated following liver injury. These activated hepatic stellate cells are primarily responsible for the excess production of extracellular matrix (Senoo et al., 1998). Thus, reduction of extracellular matrix production by activated hepatic stellate cells is crucial for the prevention of fibrogenesis. Damage to hepatocytes is the primary and continuing factor leading to hepatic stellate cell activation. Components released from the damaged cells, including lipid peroxides and reactive oxygen species, activate Kupffer cells in the liver, leading to their secretion of transforming growth factor-β (Tsukamoto, 1999). These pro-fibrogenic factors activate hepatic stellate cells, which results in liver fibrosis. Here, we showed that treatment of hepatocytes with fucoidan attenuates CCl_4_-induced cell death.

Fucoidan has been reported to interact with transforming growth factor-β and to scavenge reactive oxygen species (Boisson-Vidal et al., 1995; McCaffrey et al., 1994; Xue et al., 2001). CCl_4_ elevates serum transforming growth factor-β levels and acts as a hepatotoxin by inducing the production of reactive oxygen species (Weiler-Normann et al., 2007). In agreement with this, we have found that administration of fucoidan reduces CCl_4_-induced lipid peroxidation (data not shown). Taken together, our results suggest that the anti-fibrogenic activity of fucoidan is due, at least in part, to attenuation of hepatic stellate cell activation by inhibition of transforming growth factor-β and/or by scavenging of reactive oxygen species, which can suppress the cascade of events that leads to hepatic stellate cell activation.

Hepatocyte injury is thought to promote fibrosis, leading to the release of activators from Kupffer cells. In contrast, hepatic stellate cell apoptosis is thought to be essential for the resolution phase of fibrosis (Canbay et al., 2002; Song et al., 2003; Iredale, 2001; Iredale et al., 1998; Issa et al., 2001). Thus, a compound that can prevent hepatocyte injury and/or induce the death of hepatic stellate cells should be useful for the treatment of hepatic fibrosis. In the current studies we showed that fucoidan reduces the growth of hepatic stellate cells and that it can protect hepatocytes from injury. We also found that the fucoidan-treated hepatic stellate cells were stained with annexin V, a marker of apoptosis (data not shown). Taken together, these findings suggest that fucoidan may be useful for treating hepatic fibrosis.

Most studies on fucoidan have used a commercially available crude extract from *F. vesiculosus*, and we used the crude fucoidan in the current study. Fucoidan contains heteropolysaccharides of various kinds besides those consisting predominantly of sulfate and fucose (Nishino et al., 1994). Patel et al. found that crude commercial fucoidan was more active than the purified fucoidan at inhibiting the proliferation of vascular smooth muscle cells, and then they speculated that a specific structure in the crude fucoidan may mediate its biological activities (Patel et al., 2002). Indeed, the content of the sulfated groups in fucoidan determines its anti-proliferative and anti-coagulant activities in fibroblasts (Haroun-Bouhedja et al., 2000). The biological activities may differ among the various structures of pure fucoidan as well as the different components of crude fucoidan. Identification of the structures of fucoidan that protect hepatocytes from hepatotoxins and that inhibit hepatic stellate cell growth is needed for the development of fucoidan as an anti-fibrotic agent.

In summary, we found that fucoidan prevents hepatocyte cell death and induces the death of hepatic stellate cells in an animal model of hepatic fibrosis. Future studies will examine the molecular mechanisms of fucoidan in hepatocytes and hepatic stellate cells. This is the first report that fucoidan has anti-fibrotic activity and that it is a promising lead for the development of anti-fibrotic agents. Identification of the molecular target and the active structure of fucoidan may lead to the development of novel anti-fibrotic agents.
